# Strategies for imputation to whole genome sequence using a single or multi-breed reference population in cattle

**DOI:** 10.1186/1471-2164-15-728

**Published:** 2014-08-27

**Authors:** Rasmus Froberg Brøndum, Bernt Guldbrandtsen, Goutam Sahana, Mogens Sandø Lund, Guosheng Su

**Affiliations:** Centre for Quantitative Genetics and Genomics, Department of Molecular Biology and Genetics, Faculty of Science and Technology, Aarhus University, Tjele, 8830 Denmark

**Keywords:** Imputation, Next generation sequencing, Cross-validation, Allele frequency, Pre-phasing

## Abstract

**Background:**

The advent of low cost next generation sequencing has made it possible to sequence a large number of dairy and beef bulls which can be used as a reference for imputation of whole genome sequence data. The aim of this study was to investigate the accuracy and speed of imputation from a high density SNP marker panel to whole genome sequence level. Data contained 132 Holstein, 42 Jersey, 52 Nordic Red and 16 Brown Swiss bulls with whole genome sequence data; 16 Holstein, 27 Jersey and 29 Nordic Reds had previously been typed with the bovine high density SNP panel and were used for validation. We investigated the effect of enlarging the reference population by combining data across breeds on the accuracy of imputation, and the accuracy and speed of both IMPUTE2 and BEAGLE using either genotype probability reference data or pre-phased reference data. All analyses were done on Bovine autosome 29 using 387,436 bi-allelic variants and 13,612 SNP markers from the bovine HD panel.

**Results:**

A combined breed reference population led to higher imputation accuracies than did a single breed reference. The highest accuracy of imputation for all three test breeds was achieved when using BEAGLE with un-phased reference data (mean genotype correlations of 0.90, 0.89 and 0.87 for Holstein, Jersey and Nordic Red respectively) but IMPUTE2 with un-phased reference data gave similar accuracies for Holsteins and Nordic Red. Pre-phasing the reference data only lead to a minor decrease in the imputation accuracy, but gave a large improvement in computation time. Pre-phasing with BEAGLE was substantially faster than pre-phasing with SHAPEIT2 (2.5 hours vs. 52 hours for 242 individuals), and imputation with pre-phased data was faster in IMPUTE2 than in BEAGLE (5 minutes vs. 50 minutes per individual).

**Conclusion:**

Combining reference populations across breeds is a good option to increase the size of the reference data and in turn the accuracy of imputation when only few animals are available. Pre-phasing the reference data only slightly decreases the accuracy but gives substantial improvements in speed. Using BEAGLE for pre-phasing and IMPUTE2 for imputation is a fast and accurate strategy.

## Background

Genotype imputation is a key step in the analysis of genome-wide association studies (**GWAS**) and genomic prediction [1]. Since obtaining whole genome sequences until recently was very costly, genomic studies have mostly relied on single nucleotide polymorphism (**SNP)** marker arrays, where only a small fraction of polymorphisms are preselected to be highly polymorphic and to cover the whole genome [2]. Furthermore, it has been suggested that many quantitative trait nucleotide (**QTN**) are rare variants [3]. This means that the analyses have typically relied on linkage disequilibrium (**LD**) between markers on the SNP panel and the causal polymorphism, since QTN that affect a certain trait are not expected to be among the commonly available SNPs from marker panels. Genotype imputation allows the analysis of variants not represented in SNP arrays without the cost of genotyping millions of additional SNPs. This increases the probability that causal variants are included in the panel tested for association. Besides, with increased marker density as well as allele frequency spectrum, the chance of having higher LD among markers and QTL increases.

Recently the availability of next generation sequencing (**NGS**) techniques has made it possible to obtain whole genome sequences at a reasonable cost. The cost is however not so low that it is possible to just sequence all the individuals of interest. Therefore most of these individuals will be genotyped with SNP panels and a large part of the genomic data will have to be inferred with genotype imputation methods. In human genetics an initiative known as the 1000 Genomes project [4] has provided an open database with reference sequence data which can be used for imputing markers in a study sample. Recently an analogous initiative known as the 1000 bull genomes project was started for bovine genetics and genomics studies [5]. Currently a large part of the information in the database is dominated by Holstein bulls, but genome sequences from other dairy and beef breeds are also available.

Imputation of whole genome sequence markers offers two major challenges. 1) The accuracy of imputation depends on the size of the reference data (e.g. [6–10]), but the reference population could be small for some breeds due to the cost involved in sequencing. 2) The number of variants obtained from whole genome sequence is huge and could result in a massive computational burden.

The first problem might be overcome by pooling reference data across breeds. Previous studies on combining reference populations for imputation from 50 k to High Density (**HD**) marker panels in cattle have shown no gain in accuracy [8, 11], Brøndum et al. however concluded that the imputation accuracy could be improved for animals with a mixed genetic background without decreasing the accuracy for the purebred animals when using a combined breed reference population [6]. The limited success of across breed imputation from 50 k to HD is most likely caused by differences in LD phase and thus haplotype dissimilarity across breeds on the 50 k panel [8, 11], but the greater marker density on the HD panel might alleviate these differences for diverged breeds [12], meaning that across breed imputation could be successful from HD to whole genome sequence data [11].

The second problem could be solved by using a pre-phasing strategy. Howie et al. showed that pre-phasing the data is a time-efficient method for imputation which only results in a minor loss in accuracy [13]. A number of fast and accurate phasing approaches are available, for example, SHAPEIT2 [14] and BEAGLE [15].

It has been shown that high accuracies of imputation from 50 k to HD data in cattle can be obtained using methods that rely mostly on population LD information [16], such as BEAGLE [15] and IMPUTE2 [17]. Ma et al. showed that IMPUTE2 outperformed BEAGLE for rare variants, but at a cost of longer computation time, and that slightly higher accuracies for rare variants was possible when using Fimpute [18], which also uses pedigree information [16]; a full pedigree across countries and breeds was however not available for the data in this study. BEAGLE is not designed for an admixed reference population, but it seems that the underlying graphical model describing the patterns in LD is able to properly account for substructure in the data. The model in IMPUTE2 has been designed to use local sequence similarity to build a custom reference panel for each sample haplotype from a mixed reference population [19].

The objective of this study was to investigate how well whole genome sequence variants can be imputed from a HD SNP marker panel in Nordic dairy bulls. We compared the accuracy using both a single or multi-breed reference population, and compared the accuracy and computation time of BEAGLE and IMPUTE2 with or without pre-phased reference data.

## Methods

The whole genome sequence data used as a reference for this study is compiled from two different sources. At Aarhus University 135 bulls from the three major Nordic dairy cattle breeds, i.e. Holstein, Jersey and Nordic Reds, have been sequenced. A subset of them has been shared in the 1000 Bulls Genomes collaboration and in turn it was possible to increase the imputation reference with an additional 107 dairy bulls from *run2* of the 1000 Bull Genomes reference data. Different variant calling pipelines where used for the two datasets; these are described below. In order to reduce computation time without losing generality only markers mapped to Bovine autosome 29 (**BTA29**) were investigated. However, similar results are expected for other autosomes.

### Nordic sequence data (N = 135)

DNA was extracted from semen samples using standard procedures at Aarhus University, Foulum. Sequencing was done using Illumina sequencers at Beijing Genomics Institute, Shenzhen, China. Sequencing was shotgun paired-end sequencing with a read length of 91 base pairs. Fastq data were converted from Illumina to Sanger quality encoding using a patched version of maq [20]. They were aligned to the UMD3.1 assembly of the cattle genome [21] using bwa v0.6.2 [22]. They were converted to BAM files using samtools [23]. Quality scores were re-calibrated using the Genome Analysis Toolkit v1.6 [24] following the Human 1000 Genome guidelines incorporating information from dbSNP vers. 133 [25]. Sequence realignment around insertion/deletions (**INDEL**) and variant calling was done using the Genome Analysis Toolkit v1.6.

### 1000 Bull genomes run2 (N = 234)

Variants were called using Samtools-0.0.18. Additional custom made filters were used to remove false positive variant calls, and filters were calibrated by the concordance of sequence and Bovine High Density Chip genotypes and the rate of opposing homozygotes for sire-son paired genotypes. Detailed guidelines are available at http://www.1000bullgenomes.com.

### Combined sequence data

Data from both sources were available as **VCF** files, containing both genotype probabilities and unphased most likely alleles; for a full description of the data-format see [26]. Data was combined using Picards MergeVCFs (http://picard.sourceforge.net). From the available datasets only Holstein, Jersey, Nordic Red and Brown-Swiss bulls were kept for further analysis, leaving a total of 242 dairy bulls, with a mean sequencing depth of 13.5x as a reference for imputation. As the 1000 bull genomes project shares data after variant calling, some markers were not called for all animals in the combined dataset. To avoid large gaps of missing markers in the dataset only markers that were called in both the Nordic and the 1000 bull genomes project datasets were kept. For positions containing both a SNP and an INDEL, the INDEL was deleted as the imputation methods rely on unambiguous sequences of variants. Positions with disagreements between alleles for sequence and HD data were also deleted. Reference genotype probability data was run through BEAGLE and all markers with an R^2^ value (imputation quality at imputed marker) below 0.9 were removed from the original sequence data. This was done in order to remove uncertain marker genotypes that might have adverse effects on the imputation procedures, but might also cause a positive bias in the estimated imputation accuracies. These quality control steps left a total of 387,436 markers on BTA29: 362,122 SNPs and 25,314 INDELs.

### HD data

Seventy-two of the sequenced Nordic bulls had previously been genotyped with the Illumina Bovine HD chip (Illumina, Inc., San Diego, CA) and were chosen as a validation set. For the HD data, markers that were monomorphic, had a GenCall score of less than 0.60 or a call rate less than 0.95 were removed. These quality control steps left 13,612 markers from BTA29. HD marker data for the Nordic Holstein and Nordic Reds have previously been used in a number of studies on both genomic prediction and imputation, e.g. [6, 27, 28].

For more detail on the number of animals per breed from each data source, see Table 1.

**Table 1:** **Number of animals with whole-genome sequence and high density genotype information used in the study**

	Holstein	Jersey	RDC	Brown-Swiss	Total
**Nordic sequence**	40	27	52	16	135
**1000 bull genomes sequence**	92	15	0	0	107
**High density SNP data**	16	27	29	0	72

### Imputation

Imputation accuracy from HD to sequence data was tested in a number of different scenarios for comparison on both imputation accuracy and speed. Pre-phasing of the reference data was done using either BEAGLE v3.3.2 or SHAPEIT2 v2.644, both with default parameters and using genotype probability data as input.

Imputation was done in BEAGLE or IMPUTE2 using either pre-phased haplotypes or genotype probabilities. For all scenarios a leave-one-out validation was done, where each of the animals with both sequence and HD data in turn was deleted from the reference data and included as a target individual with only HD data. Accuracy was computed as the correlation of the true genotypes from sequence calls and imputed dosages.

### BEAGLE

For BEAGLE two different scenarios were tested. The first was the effect of using a combined reference population versus using a single breed reference population; the second was using pre-phased reference data from either BEAGLE or SHAPEIT2 versus using un-phased genotype probability data. The first scenario was only tested using pre-phased reference data. A previously designed pipeline with BEAGLE where the data was divided in chunks of ~20,000 markers with an overlap of 500 markers was used for the imputations with phased reference data. The number of HD markers in these chunks averaged 707 with a range from 402 to 928. Imputations with un-phased data were done for the entire chromosome in one run. All imputations were done using the default parameters.

### IMPUTE2

For IMPUTE2 we did not test the effect of single- versus multi-breed references; only the effect of pre-phased most likely genotypes versus genotype probability data was investigated. Pre-phased data was obtained from either BEAGLE or SHAPEIT2. The imputations were done in chunks of 2.75 MB (giving the same number of chunks as in BEAGLE). The number of sequence markers in these chunks including buffer regions of 0.25 MB averaged 24,000 with a range from 10,000 to 35,000, and the number of HD markers averaged 800 with a range from 357 to 1039. The effective population size was set to 100 and all other parameters were kept at their default values.

## Results and discussion

The quality of the sequence data was assessed by comparing best calls from the sequence data with variants from the HD chip for the 72 test animals. Results showed a mean concordance of 99.32%, with a range from 97.43% to 99.93%. The mismatches between HD and sequence data suggests that the best calls based solely on individual sequence information, does not necessarily represent the actual variants, which might cause a negative bias in the assessment of imputation accuracy. It was found that the concordance between HD and sequence variants increased to an average of 99.86% with a range from 99.16% to 99.96% when the comparison was done based on best calls from BEAGLE posterior genotype probabilities instead of using the genotype probabilities obtained from the variant calling software, which strengthens this hypothesis. All calculations of accuracy below are however done based on raw genotype calls to avoid a bias in favor of either BEAGLE or IMPUTE2.

Mean imputation accuracies for the three breeds are shown in Table 2. By comparing the first and second row of the table it is seen that all populations gain from using the combined reference population, with the largest gain for Nordic Reds. These results are in line with previous results by Brøndum et al. [6], where it was found that the imputation accuracy for Danish Reds was improved when including Holsteins in the reference. All of the Nordic Red animals included as test animals are from Danish Red which historically has used sires from both Holstein and Brown-Swiss. Furthermore, the accuracy of imputation has been shown to depend on the relationship between reference and validation animals [7]. An investigation of the available pedigree data showed that the for the RDC validation animals there were 1 sire, 1 maternal grandsire and 7 paternal grandsires in either the Holstein or Brown-Swiss reference data. These facts combined could explain the large gains in accuracy for the RDC test animals. Holstein animals gain very little from inclusion of other breeds, which could be due to their relatively large single breed reference population. For the Jerseys an investigation of the available pedigree data showed no relationships between test animals and the added animals in the combined reference population. Furthermore, a principal component analysis of the Nordic dairy breeds, shows that the Jersey population and the other populations analyzed in this study are quite distinct with no evidence of recent admixture [29]. The small gain in imputation accuracy for the Jersey population when using the combined reference population thus suggests that the persistence of LD phase between the HD markers across populations is strong enough to utilize information from one population in the other for imputation from HD to full sequence data, even with distant relations.

**Table 2:** **Mean and standard deviation (SD) of correlation between true and imputed genotype dosage for Holstein (HOL), Jersey (JER) and Nordic Red (RDC)**

METHOD	HOL		JER		RDC	
	Mean	SD	Mean	SD	Mean	SD
BEAGLE/BEAGLE pre-phasing (Single breed)	0.87	0.32	0.82	0.38	0.76	0.39
BEAGLE/BEAGLE pre-phasing	0.88	0.32	0.87	0.32	0.86	0.30
BEAGLE/SHAPEIT2 pre-phasing	0.88	0.30	0.86	0.32	0.85	0.30
BEAGLE/Genotype probabilities	0.90	0.27	0.89	0.28	0.87	0.27
IMPUTE2/BEAGLE pre-phasing	0.90	0.20	0.85	0.23	0.86	0.20
IMPUTE2/SHAPEIT2 pre-phasing	0.90	0.20	0.84	0.23	0.86	0.21
IMPUTE2/Genotype probabilities	0.90	0.20	0.84	0.22	0.87	0.18

Results for the comparison between BEAGLE and IMPUTE2 with combined reference populations are also shown in Table 2. Overall the accuracies from different scenarios are quite similar within the respective breeds, but across the three breeds the highest imputation accuracies are found when using BEAGLE with un-phased reference data. Similar accuracies are found when using IMPUTE2 for the Holstein and RDC animals, but for the Jersey animals BEAGLE with un-phased reference data has the best results. For BEAGLE, there seems to be some loss of accuracy when using pre-phased reference data, but IMPUTE2 is less affected. The larger difference for BEAGLE, could be caused not only by the differences in input data, but also by dividing the data in smaller chunks in the phased setup, although an overlap between chunks was used to avoid increases in error rate at either end. IMPUTE2 also uses a strategy to improve speed by dividing the data in smaller chunks, but the number of overlapping markers was larger. When comparing accuracies obtained using pre-phased data, a slight advantage is seen for pre-phased data from BEAGLE in both BEAGLE and IMPUTE2, although SHAPEIT2 is the recommended software for pre-phasing for IMPUTE2. Possibly better results could be obtained by increasing the number of conditioning states in the SHAPEIT2 algorithm, but this would also result in increased computation time, and it has previously been shown that the gain is limited when exceeding the default value of 100 states [14].

**Figure 1:**
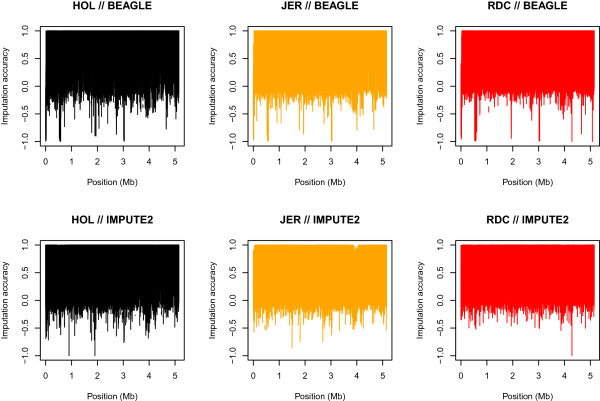
**Imputation accuracy along BTA29 for Holstein (HOL), Jersey (JER) and Nordic Red (RDC).** Imputed data was obtained using BEAGLE or IMPUTE2 with genotype probability data in the reference.

Figure 1 shows the imputation accuracy across BTA29 for the two most accurate scenarios, i.e. BEAGLE and IMPUTE2 with genotype probability data and a combined reference population. There is a large variation with accuracies ranging from -1 to +1, i.e. from opposite imputation to completely true. Spikes of low imputation accuracy are consistent across the three breeds in BEAGLE which might result from problems in sequencing these regions or from errors in the assembly since the imputation procedures rely on correct sequences of markers. Comparing the results from BEAGLE to results from IMPUTE2 there seems to be some overlap in spikes of low accuracy; they are, however, less pronounced for IMPUTE2.

**Figure 2:**
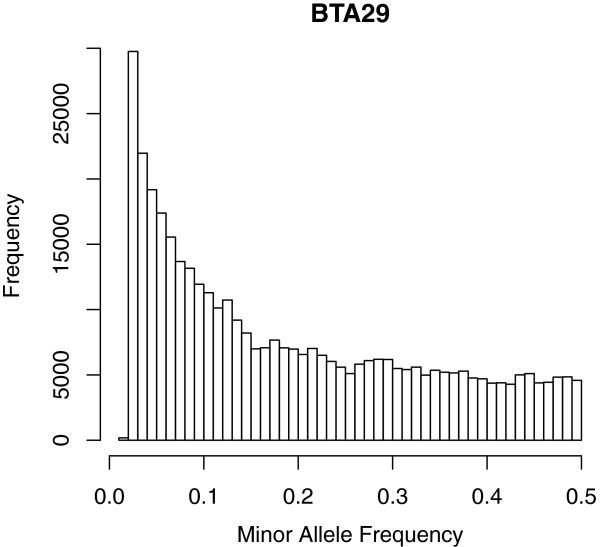
**Distribution of minor allele frequency for sequence markers on BTA29.** Minor allele frequencies are calculated based on the 242 sequenced animals.

**Figure 3:**
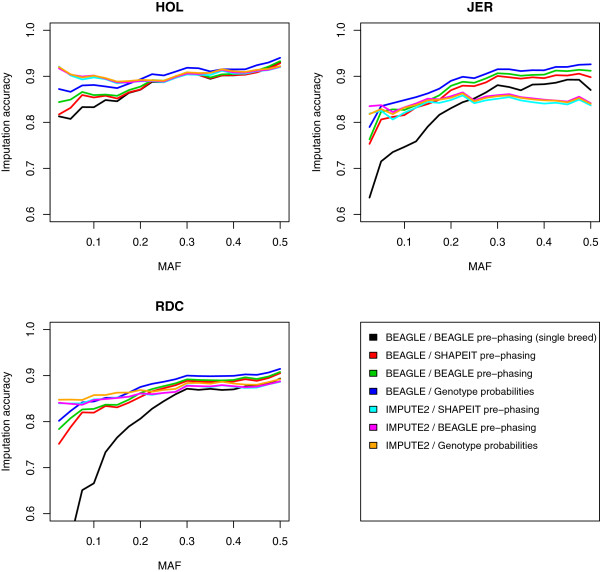
**Imputation accuracy versus minor allele frequency (MAF).** Imputation accuracies are averaged in bins of 1% of MAF.

One of the powers in sequence data lies in the ability to use rare variants for analysis, and these have to be imputed accurately in order to use them efficiently. In other words it is important that the imputation procedure performs well for the full allele frequency spectrum. Figure 2 shows that the distribution of loci across the minor allele frequency (MAF) spectrum is skewed to the left which means that poor imputation for rare variants would heavily influence the overall imputation accuracies. On Figure 3, average imputation accuracies are shown plotted against the MAF; averages where calculated in bins of 1%. This was done for each of the seven scenarios listed in Table 2. Results show that IMPUTE2 performs better than BEAGLE when imputing rare variants, which confirms previous results [16], but on the contrary BEAGLE performs better for the more common variants . This larger variation in accuracy across the allele frequency spectrum when using BEAGLE is also evident from the larger standard deviations for BEAGLE in Table 2. In addition, for BEAGLE the pre-phased setups seem to have the largest impact on the imputation error for loci with low MAF, whereas this pattern is less noticeable for IMPUTE2. For the Jerseys and Nordic Reds imputation accuracies drop fast for MAF less than 0.3 in the single breed reference scenarios. This might be caused by the fact that many rare variants would be poorly represented in the single breed imputation references for the smaller breeds. Looking at the overall picture it seems that with the current size of the imputation reference, there is not enough information for accurate imputation of rare alleles, but the reference is growing, and the accuracy is expected to follow. However, it might also be possible to increase the accuracy for rare variants with the current size of reference data by using methods such as Fimpute that utilize pedigree data along with the LD information as shown in the results by Ma et al. [16].

The accuracies of imputation from HD to whole genome sequence data obtained in this study are larger than previous results from the 1000 bull genomes project where an average accuracy of imputation (correlation of true and imputed allele dosage) of 0.80 was reported when using BEAGLE within the Holstein breed only [5]. These results where however obtained using a validation procedure that removed more animals from the reference. Accuracies of imputation to whole genome sequence are much lower than those reported for imputation from 50 k to HD data in cattle with BEAGLE and IMPUTE2. Genotype correlations of 0.97 have been reported for imputation of HD markers from 50 k in Nordic Holstein and Red [6, 16], and Berry et al. reports genotype correlations of 0.98 and above for imputation from 50 k to HD data in 7 different dairy and beef breeds [11]. The higher accuracies obtained in these studies are most likely caused by larger within breed references and fewer markers with very low minor allele frequencies.

Computation times for the different procedures are shown in Table 3. It is seen that with un-phased reference data computational demands for IMPUTE2 are much higher than what is required for BEAGLE with un-phased reference data (42 hours vs. 3 hours per individual). When using pre-phased reference data IMPUTE2 is however, much faster than BEAGLE (5 minutes vs. 50 minutes per individual).

**Table 3:** **Computation times for phasing and imputation procedures**

Procedure	Approximate CPU time (hour:min)
Phasing reference (N = 242)	
BEAGLE	02:30
SHAPEIT2 (4 cores)	52:00
Imputing one individual (ref: N = 241, validation: N = 1)	
BEAGLE with phased reference	00:50
BEAGLE with un-phased reference	02:50
IMPUTE2 with phased reference	00:05
IMPUTE2 with un-phased reference	41:40

Computations were done on a Unix computer cluster with Intel XEON X5670/X5677 processors.

For the phasing procedures BEAGLE was faster, than SHAPEIT2 which required around 52 hours to phase BTA29 for the 242 available animals, whereas BEAGLE performed the same job in less than 3 hours. SHAPEIT2 supports parallel computing, and this implementation has been shown to be competitive with BEAGLE and in fact faster for sample sizes of 3,000 individuals or more using a SNP density similar to the bovine HD panel [14]. In the data at hand we were not able to reproduce this result, which suggests that for smaller sample sizes BEAGLE is much more effective; there might however also be an effect of the SNP density, but a more in depth investigation is needed to confirm this. Furthermore BEAGLE 4 [30] which was recently released also supports parallel computation, which presumable makes the advantage larger.

All estimates of imputation runtimes were done using only one test individual. To validate that the ranking of methods also holds for larger samples sizes we reran a single chunk of 20,000 markers using pre-phased reference data with the 242 sequenced animals in the reference and 2,000 animals with HD data. We found that BEAGLE was able to impute all these animals in 8 hours, whereas IMPUTE2 required only 4 hours. If the HD data was also pre-phased, BEAGLE was able to complete the task in 1 hour, whereas IMPUTE2 used only 10 minutes. Using the pre-phased HD data did not decrease the imputation accuracies (results not shown), but further studies are required to confirm these results as they include information from many more HD animals in the pre-phasing which could improve the phasing quality, thus making them not directly comparable. A previous study by Howie et al., however shows that pre-phasing the study data only results in a minor loss of imputation accuracy [13].

## Conclusion

When imputing whole genome sequence variants with a limited number of reference individuals, combining the references across breeds is a good strategy to improve the imputation accuracy. Furthermore, IMPUTE2 is more accurate than BEAGLE for rare variants. Using IMPUTE2 with pre-phased data from BEAGLE is computationally efficient, and only results in a minor loss in accuracy. Overall accuracies are lower than previous reports on imputation of HD markers, especially for rare alleles. There is also a large variation in the accuracy across loci, so the added benefit of sequence data for genomic prediction at the moment might be limited. Imputation accuracy is, however, expected to improve as the size of the reference population increases. Taking both computation time and accuracy into account, using BEAGLE for pre-phasing and IMPUTE2 for imputation would be a good strategy for large scale imputation of whole genome sequence markers.

### Availability of supporting data

All DNA sequences used were taken from a publicly available assembly. The assembly is available for download at ftp://ftp.ensembl.org/pub/release-73/fasta/bos_taurus/dna. All variations identified have been submitted by the 1000 Bull Genomes project for inclusion in dbSNP (http://www.ncbi.nlm.nih.gov/SNP). Whole genome sequence data for the 234 individuals included in run2 of the 1000 bull genomes project are available at NCBI using SRA no. SRP039339 (http://www.ncbi.nlm.nih.gov/bioproject/PRJNA238491).
